# Delayed progression of prion disease in mice by polyarginine-facilitated prevention of PrP^Sc^ propagation in the spleen

**DOI:** 10.1016/j.neurot.2025.e00560

**Published:** 2025-02-26

**Authors:** Sungeun Lee, Jieun Kim, Yoonjeong Lee, Miryeong Yoo, Jaehyeon Kim, Hyun Joo Sohn, Chongsuk Ryou

**Affiliations:** a Department of Pharmacy, College of Pharmacy, and Institute of Pharmaceutical Science & Technology, Hanyang University ERICA, 55 Hanyangdaehak-ro, Ansan, Gyeonggi-do, 15588, Republic of Korea; b Foreign Animal Disease Division, Department of Animal and Plant Health Research, Animal and Plant Quarantine Agency, 177Hyeoksin 8-ro, Gimcheon-si, Gyeongsangbukdo, 39660, Republic of Korea

**Keywords:** Prions, Prion diseases, Poly-l-arginine, Spleen, Follicular dendritic cell

## Abstract

Prions are infective agents composed of abnormally folded prion proteins (PrP^Sc^), which are pathogenic isoforms of normal cellular prion proteins (PrP^C^) that cause incurable, transmissible, neurodegenerative conditions in mammals called prion diseases. The spread of PrP^Sc^ within a host is facilitated by the lymphoreticular system, which uptakes and propagates PrP^Sc^ in the periphery and transmits them to the central nervous system. Our previous study showed that poly-l-arginine (PLR), a cationic amino acid polymer, inhibits PrP^Sc^ accumulation in neuroblastoma cells with persistent prion infection (ScN2a). Here, we report the beneficial effect of PLR against prions. In the *in vitro* prion infection experiment, PLR efficiently reduced the titer of prions inoculated to infect cultured N2a cells. In animal experiments, PLR inhibited the accumulation of PrP^Sc^ in the spleens of mice intraperitoneally inoculated with prions during asymptomatic periods. Prophylactic administration of PLR significantly prolonged incubation periods in mice intraperitoneally infected with prions, mitigating vacuolation and astrogliosis, although PrP^Sc^ level was not dramatically reduced in the brain. However, PrP^Sc^ level was reduced and the marginal zone distortion associated with prion infection was prevented in spleens of mice that was intraperitoneally infected with prions and received PLR, even at the terminal stage. Expression of follicular dendritic cell (FDC)-M1 antigens, a marker of FDC activation, and the level of PrP^Sc^ colonized within the white pulp of the spleens, as well as co-localization of FDC-M1 antigens and PrP^Sc^, were reduced in these mice during the course of disease, suggesting that PLR counteracts the ability of FDCs that support PrP^Sc^ propagation in the spleen. Overall, prophylactically administered PLR suppresses prions *in vivo*, presumably through cellular control of pathological processes that occur in the spleen and eventually delay prion spread to the brain. This study presents implications for modulating the progress of prion diseases acquired peripherally.

## Introduction

Prion disease, also known as transmissible spongiform encephalopathy, is characterized by the accumulation of scrapie prion proteins (PrP^Sc^), the disease-associated isoform of the cellular prion proteins (PrP^C^), in the periphery and central nervous system (CNS) during disease progression [[1], [2], [3]]. As it progresses, prion disease causes neuronal cell death, astrogliosis, and vacuolation, leading to spongiform degeneration in the brain [1,4,5].

Some cases of prion disease occur spontaneously, while others are caused by exposure to externally derived prions through both direct neuronal contact and interactions between the nervous and immune systems [4,[6], [7], [8], [9]]. When prions infect genetically immune-deficient [8,10] and surgically [11] or pharmacologically [12,13] splenic function–restricted mice through the peripheral routes, disease progression is delayed or prion transmission fails. The microfold (M) cell of Peyer’s patch initially takes up orally acquired prions from the gut lumen and then prions are transferred to the lymphoreticular system (LRS) [14,15]. The spleen, which is a part of the LRS, plays a role in prion proliferation and transport from the periphery to the CNS [16,17]. Upon initial prion infection through the peripheral routes, follicular dendritic cells (FDCs) in the germinal center of the spleen play a pivotal role in PrP^Sc^ multiplication [13,18]. FDCs express PrP^C^ that can be converted to PrP^Sc^ [19,20], which are then secreted from FDCs and accumulate in the white pulp of the spleen [21]. Prions amplified in the spleen spread to the CNS through the mid-thoracic spinal cord via visceral sympathetic neurons [[22], [23], [24], [25]]. Amplification of PrP^Sc^ can be detected in the LRS prior to CNS invasion.

To date, prion diseases remain incurable. Many agents, including quinacrine, pentosan polysulfate, flupirtine, and doxycycline, reportedly exhibit efficacy in some *in vitro* and *in vivo* models but have failed in human trials [[26], [27], [28], [29], [30], [31], [32]]. Anti-PrP antibodies and antisense oligonucleotides that hinder prion disease *in vitro* and *in vivo* are under evaluation in human patients [33,34]. Among other effective agents, cationic polymers have demonstrated anti-prion efficacy in some studies [[35], [36], [37], [38]]. One of our previous studies demonstrated that poly-l-lysine decreases PrP^Sc^ level in ScN2a cells and delays disease onset in mice intracraneally infected with scrapie prions [39]. We also showed that poly-l-arginine (PLR), another cationic amino acid polymer, inhibits PrP^Sc^ propagation in ScN2a cells with a greater potency than other amino acid polymers [40,41]. Furthermore, intracellular assembly of nanoparticles displaying oligo-arginines effectively inhibits PrP^Sc^ propagation in ScN2a cells, avoiding the potential cytotoxicity of bulky PLR [42].

Encouraged by the ability of PLR and oligo-arginines to inhibit PrP^Sc^ propagation in ScN2a cells, we investigated the anti-prion efficacy of PLR in animal models. The etiology of prion diseases is sporadic in most cases, but among those caused by infection with externally derived prions, variant Creutzfeldt-Jakob disease (vCJD) and some cases of iatrogenic Creutzfeldt-Jakob disease CJD (iCJD) are caused by indirect infection to non-CNS tissues rather than direct infection within the brain [43,44]. To cope with the cases caused by peripheral prion infection such as oral exposure to prions in vCJD and peripheral exposure to prions in iCJD *via* hormone therapy, blood transfusion, and corneal transplantation, we evaluated the efficacy of PLR using a prion disease model with peripheral infection, in which a preventive approach to suppressing the disease before prions invade the CNS is feasible. In the current study, the effects of PLR on the disease course, suppression of PrP^Sc^ propagation, and prevention of pathological alterations in the spleen and brain were examined using tissues from a mouse model of prion disease. We particularly focused on the alteration in splenic tissues and cells with respect to the effect by PLR to determine how PLR modifies prion disease progress at the cellular level.

## Materials and methods

### Chemicals

The chemicals used in the study were purchased from Sigma-Aldrich (St. Louis, MO, USA), unless stated otherwise.

### Standard scrapie cell assay

Standard scrapie cell assay (SSCA) was performed according to methods described elsewhere with minor modifications [45]. Briefly, 2.4 ​× ​10^4^ N2a cells cultured in a 24-well dish were incubated with 0.3 ​% (w/v, final concentration) brain homogenate prepared in phosphate buffered saline (PBS, Thermo Fisher Scientific, Waltham, MA, USA) from mice that were terminally ill due to infection with mouse-adapted scrapie RML prions (hereafter RML-sick brain homogenate [SBH]) for four days [39]. The PLR at a non-cytotoxic concentration [40] (40 ​nM of PLR hydrochloride, 200 repeating units, molecular weight of 38.5 ​kDa; Alamanda Polymers, Huntsville, AL, USA) was administered either immediately after or one day prior to prion inoculation. Incubation with PLR lasted for four days for the former and for one day for the latter. The cells were cultured in prion- and PLR-free media for multiple passages. As a control, non-infected N2a cells were also grown. At the tenth passage, 2.5 ​× ​10^4^ ​cells were seeded and fixed on an activated opaque 96-well plate with a 0.45 ​μm hydrophobic high-protein-binding Immobilon-P membrane (Merck Millipore, Darmstadt, Germany). The cells were incubated in a lysis buffer (50 ​mM Tris, 150 ​mM sodium chloride, 1 ​% sodium deoxycholate, 1 ​% Nonidet P-40, and 0.1 ​% sodium dodecyl sulfate [SDS]) with 5 ​μg/mL proteinase K (PK, Roche, Basel, Switzerland) for 90 ​min and then in PBS with 2 ​mM phenylmethylsulfonyl fluoride (PMSF, Merck Millipore, Darmstadt, Germany) for 20 ​min. The membrane was soaked in denaturation buffer (3 ​M guanidine thiocyanate in PBS) for 10 ​min and rinsed carefully with PBS. PrP^Sc^ was selectively labeled with mouse monoclonal anti-PrP antibody 6D11 (1:10,000 diluted, Biolegend, San Diego, CA, USA) and alkaline phosphatase-conjugated mouse immunoglobin G (IgG; 1:5000 diluted, Abcam, Cambridge, UK) after blocking with Superblock solution (ThermoFisher Scientific, Waltham, MA, USA). The number of infected cells was counted using an S6 universal M2 ELISPOT reader (ImmunoSpot, Shaker Heights, OH, USA) and Immunospot software (ImmunoSpot, Shaker Heights, OH, USA) after color development using a nitro-blue tetrazolium chloride/5-bromo-4-chloro-3ʹ-indolyphosphate p-toluidine salt substrate.

### Animals

All experimental procedures involving animals were approved by the Institutional Animal Care and Use Committee at Hanyang University, Seoul, Republic of Korea (2017-0158A). The animal experiments followed methods described previously [39]. The prion disease model was established by inoculating five-week-old female CD-1 mice (Samtaco, Osan, Korea) with 50 ​μL of 1 ​% (w/v) RML-SBH through an intraperitoneal (IP) route.

To determine the anti-prion activity of PLR in the spleens of RML prion–inoculated mice at an asymptomatic stage, 25 ​mg of PLR per kg of body weight was administered intraperitoneally twice a week for eight weeks beginning one day after RML-SBH inoculation (day post-infection [dpi] 1). PBS was used as the vehicle for the control groups. The spleens were collected at 84 dpi and stored at −80 ​°C until they were used for western blotting for PK-resistant PrP^Sc^.

The *in vivo* anti-prion efficacy of PLR was determined by measuring the delay of disease onset in RML prion–inoculated mice. The details of the prion inoculation route, PLR administration route, frequency and duration, and PLR dosages are summarized in Table S1. For the suppression experiments, PLR was administered one day after prion inoculation through an IP route. For the prevention experiment, PLR was administered to three-week-old mice once a week for two weeks prior to RML-SBH inoculation. All experiments included a PBS vehicle group that served as a control. At the terminal stage, when mice exhibited clinical signs associated with prion disease, such as ataxia, weight loss, kyphosis, and tail rigidity, they were humanely euthanized, and the tissues and organs were collected. Collected brains and spleens were either cryopreserved for western blotting or fixed in 4 ​% paraformaldehyde for pathological analysis. Fixed mouse brains and spleens were used to prepare paraffin blocks using a Citadel 2000 tissue processor (ThermoFisher Scientific, Waltham, MA, USA).

### Western blotting

Levels of PrP^Sc^ in the brain and spleen were determined using western blotting by a previously described method [39]. The tissue homogenate (10 ​% w/v) was prepared using an OMNI Bead Ruptor 24 (PerkinElmer, Shelton, CT, USA) in PBS. The concentrations of proteins were measured using a Pierce bicinchoninic acid protein assay kit (ThermoFisher Scientific, Waltham, MA, USA) after centrifugation at 1000 ​*g* for 5 ​min at room temperature. Brain homogenate (0.2 ​mg protein) or spleen homogenate (1 ​mg protein) in 2 ​% Sarkosyl-PBS was incubated with 20 ​μg/mL PK for 1 ​h at 37 ​°C. The reaction was stopped by 2 ​mM PMSF and then centrifuged at 16,000 ​*g* for 1 ​h at 4 ​°C. The pellet was dissolved in sample loading buffer (2 ​% SDS, 10 ​% glycerol, 0.002 ​% bromophenol blue, 62.5 ​mM Tris-HCl, pH 6.8, 5 ​% β-mercaptoethanol), separated in a 15 ​% SDS-acrylamide gel, and electro-transferred to an Immobilon-P membrane (Merck Millipore, Darmstadt, Germany). After blocking with 5 ​% Difco skim milk (BD, Franklin Lakes, NJ, USA) in Tris-buffered saline containing 0.1 ​% Tween-20 for 1 ​h at room temperature, the membrane was incubated with mouse monoclonal anti-PrP antibodies 6D11 (1:30,000 diluted) at 4 ​°C overnight and subsequently with horseradish peroxidase (HRP)-conjugated goat anti-mouse IgG antibodies (1:10,000 diluted, ThermoFisher Scientific, Waltham, MA, USA) for 1 ​h at room temperature. The PK-resistant PrP^Sc^ bands were detected using the Amersham ECL Prime western blotting detection reagent (Cytiva, Marlborough, MA, USA), and the signal was obtained using the Gbox Chemi XR5 image processing system (Syngene, Cambridge, UK). To measure levels of β-actin, total PrP, and FDC-M1 (an FDC marker), 20 ​μg protein of brain or spleen homogenate in 2 ​% Sakosyl-PBS without PK digestion was used for western blotting. Mouse monoclonal anti–β-actin (1:5000 diluted, Santa Cruz Biotechnology, USA), rat monoclonal anti-mouse FDC-M1 (1:500 diluted, BD Pharmingen, Franklin Lakes, NJ, USA), and mouse monoclonal anti-PrP 6D11 (1:30,000 diluted) antibodies were used as the primary antibodies. The same secondary antibody described in PrP^Sc^ western blotting was used to detect β-actin and total PrP. For FDC-M1 detection, biotinylated anti-rat IgG (1:500 diluted; Vector Laboratory, Newark, CA, USA) and streptavidin-HRP (1:10,000 diluted; Abcam, Cambridge, UK) were used to incubate the blot sequentially. Densitometry was carried out using GeneTools software (Syngene, Cambridge, UK).

### Histology and lesion profiling

Paraffin-embedded tissues were cut into five-μm-thick coronal slices using an HM 340E microtome (ThermoFisher Scientific, Waltham, MA, USA). To confirm changes in histology, brain and spleen blocks were deparaffinized, rehydrated, and stained with Mayer’s hematoxylin and Eosin Y (H&E, Cancer Diagnostics, Durham, NC, USA). Images of the stained tissue were obtained using a Virtual Microscope Axioscan Z1 tissue scanner (Zeiss, Oberkochen, Germany) and ZEN 2.3 software (Zeiss, Oberkochen, Germany). The degree of vacuolation in the brain was determined by counting the number of vacuoles within an arbitrary unit area of tissue sections using ZEN 2.3 (Zeiss, Oberkochen, Germany) or ImageJ (NIH, Bethesda, MD, USA). The arbitrary unit area with a consistent dimension (605 ​μm width ​× ​405 ​μm height) equals to 0.245 ​mm^2^ and was selected randomly from scanned images. Scoring of the splenic marginal zone (MZ) distortions was performed by the methods described elsewhere [46]. The scoring criteria was the irregularity of the MZ boundary facing the mantle zone of white pulp and the red pulp. The transected spleen was stained with H&E, and images of the central region of the spleen including white pulp and surrounding red pulp were obtained as described earlier. Six independent spots from a spleen were randomly chosen and subjected to blind analysis for MZ scoring. MZ interface distortion was determined by estimating the percent of MZ circumference involved in protrusion toward the center of the white pulp. Distortions of 75 ​% or greater interface received a rating of 0, 50%–75 ​% distortions a 1, and 12.5%–50 ​% distortions a 2. Partial distortions of less than 12.5 ​% received a 3, and a lack of distortion a 4. The average score was obtained from three independent researchers, including a pathologist, without prior information about the sample. The MZ scores were confirmed by a pathologist.

### Immunohistochemistry

Coronal brain sections and transections of the spleen were sliced at 5 ​μm and hydrated after deparaffinization. Hydrated tissues were boiled in 10 ​mM sodium citrate buffer (pH 6.0) for 10 ​min before incubation with 3 ​% hydrogen peroxide for 5 ​min at room temperature. For detection of PrP^Sc^, incubation of tissue slices with 10 ​μg/mL PK in PBS for 10 ​min at 37 ​°C was carried out between boiling and oxidation. For detection of PrP^Sc^ and glial fibrillary acidic protein (GFAP), the tissue slices were blocked with a blocking reagent in an MOM kit (PK-2200; Vector Laboratories, USA) for 1 ​h at room temperature. For detection of FDC-M1, the tissue slices were blocked with 2.5 ​% normal goat serum (Vector Laboratories, Newark, CA, USA). For PrP^Sc^ immunohistochemistry (IHC), the tissue slices were incubated with mouse monoclonal anti-PrP antibody 6D11 (1:3000 diluted) for two days at 4 ​°C followed by detection with biotinylated goat anti-mouse secondary antibody (1:200 diluted) in an MOM kit. For IHC of GFAP and FDC-M1, mouse monoclonal anti-GFAP clone G-A-5 (1:500 diluted) and rat monoclonal FDC-M1 (1:250 diluted) antibodies were applied to the tissue slices for 1 ​h at room temperature. This was followed by detection with biotinylated goat anti-mouse and anti-rat secondary antibodies (1:200 diluted, Vector Laboratories, Newark, CA, USA), respectively. A non-specific reaction was examined by replacing the primary antibody with the blocking reagent. Avidin-coupled peroxidase (VECTASTAIN Elite ABC-HRP kit, Vector Laboratories, Newark, CA, USA) was used to conjugate the biotinylated secondary antibodies. The signal was developed using a 3, 3–diaminobenzidine (DAB)-peroxidase substrate (SK-4100, Vector Laboratories, Newark, CA, USA). Mayer’s hematoxylin was used for counterstaining. PBS was used for washing between steps. All images were acquired using ECLIPSE Ti microscopes (Nikon, Tokyo, Japan) or an Axioscan Z1 tissue scanner. Quantification of the effect of PLR against propagation of PrP^Sc^ and activation of FDCs in mouse spleens, and astrogliosis in astrocytes was conducted by counting the number of DAB-stained PrP^Sc^, FDC-M1, and GFAP-positive cells in the randomly chosen white pulps (*n* ​= ​3) of mouse spleens (*n* ​= ​3) by HDAB color deconvolution plugin in Image J software (v1.54). The blinded choice of spleen follicles or brain regions to be analyzed was made at three sites an organ collected from three mice of each vehicle- or PLR-administered group.

### Immunofluorescence

The 5-μm-thick spleen tissue slices were deparaffinized and incubated with 96 ​% formic acid for 2 ​min, followed by boiling in 1 ​mM EDTA (pH 8.0) for 20 ​min to detect PrP^Sc^ immunofluorescence (IF). IF staining for FDC-M1 omitted the incubation with formic acid, whereas the other steps were unchanged. The tissue slices were incubated with a Trublack Lipofuscin autofluorescence quencher (Biotium, Fremont, CA, USA) for 30 ​s to eliminate autofluorescence, followed by 5 ​% normal goat serum for 1 ​h for blocking. IF was conducted using the same primary antibody condition used for IHC. Goat anti-mouse IgG (H ​+ ​L) cross-adsorbed secondary antibodies, Alexa Fluor 488 (ThermoFisher Scientific, Waltham, MA, USA), and goat anti-rat IgG (H ​+ ​L) cross-adsorbed secondary antibodies, Alexa Fluor 647 (ThermoFisher Scientific, Waltham, MA, USA) secondary antibodies were applied for 1 ​h to detect PrP^Sc^, and FDC-M1, respectively. A DAPI solution (1 ​μg/mL) (ThermoFisher Scientific, Waltham, MA, USA) was used for counterstaining. IF images were obtained with an Olympus FV3000/FW30-ILSW confocal microscope and FV31S-SW software (ver. 2.4.1, Olympus, Tokyo, Japan). FV31S-SW viewer software was used to analyze the images (ver. 2.6, Olympus, Tokyo, Japan).

### Statistical analysis

The statistical significance of the difference in mean incubation period between groups was assessed using a two-sided log-rank test (SPSS, IBM, Armonk, NY, USA). Other statistical analyses were performed using ANOVA with Tukey method for post-hoc comparisons (SPSS). Significance was defined as a *p*-value less than 0.05 (∗), 0.01 (∗∗), or 0.001 (∗∗∗).

## Results

### PLR decreases *in vitro* prion infection

**Fig. 1:**
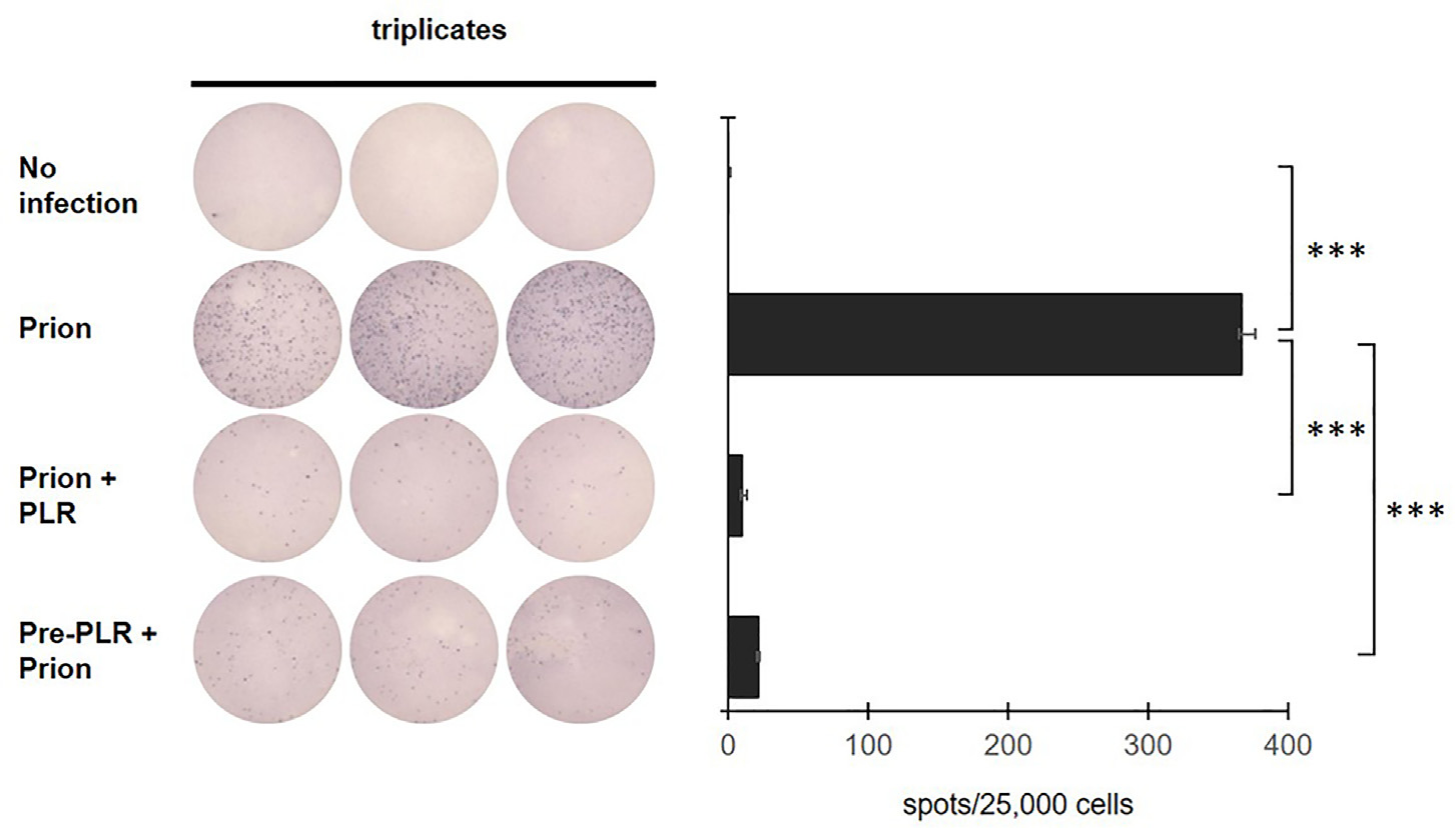
**Reduction of prion infectivity by PLR.** SSCA was performed in triplicate with RML-SBH as the prion source. The cells were inoculated with prions and then incubated with PLR (40 ​nM) for the “prion ​+ ​PLR” group. Alternatively, the cells were incubated with PLR (40 ​nM) prior to prion inoculation for the “pre-PLR ​+ ​prion” group. PrP^Sc^ was detected at passages 10 after prion infection. Stained PrP^Sc^ spots after PK digestion were counted as positives. ∗∗∗, *p* ​< ​0.001.

Inhibition of prion infection by PLR was investigated by SSCA. The N2a cells inoculated with RML-SBH yielded nearly 400 PrP^Sc^-positive spots, while the cells with no infection showed no such spots (Fig. 1). N2a cells inoculated with RML-SBH and subsequently incubated with PLR at a non-cytotoxic concentration (40 ​nM) [40] formed nearly 95 ​% fewer PrP^Sc^-positive spots. Similarly, N2a cells pre-incubated with 40 ​nM PLR for a day and inoculated with RML-SBH also yielded approximately 90 ​% fewer PrP^Sc^-positive spots. Efficient prevention of *in vitro* prion infection indicates that PLR reduces the tier of prions used to inoculate N2a cells.

### PLR inhibits PrP^Sc^ accumulation in the spleen during incubation period

**Fig. 2:**
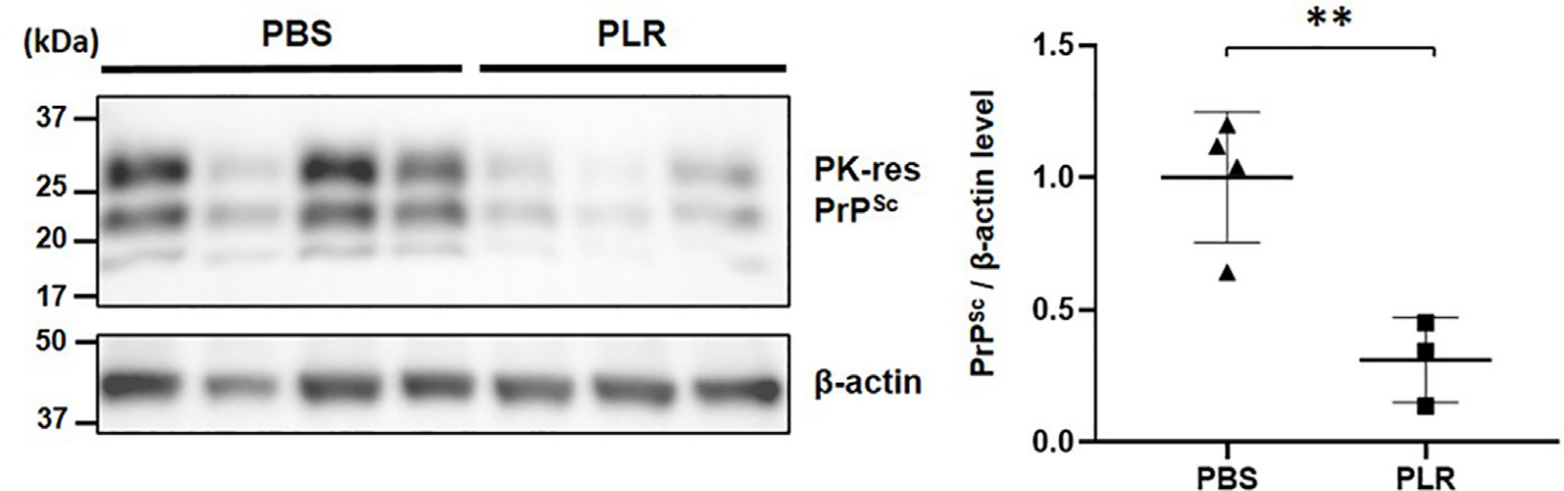
**Reduction of PrP**^**Sc**^**accumulation by PLR in the spleens of prion-infected mice during the asymptomatic period.** Groups of wild-type mice were IP inoculated with RML-SBH and IP administered PLR once a week for eight weeks [PBS vehicle group (▲), *n* ​= ​4; 25 ​mg/kg PLR group (▪), *n* ​= ​3]. The spleen was collected at 84 dpi, and PK-resistant PrP^Sc^ of spleen homogenate was analyzed by western blotting. The level of PrP^Sc^ was quantified by densitometry and normalized to β-actin level. ∗∗∗, *p* ​< ​0.001.

To address the effect of PLR in tissues experiencing initial prion propagation during asymptomatic periods of prion disease, PrP^Sc^ levels were determined by western blotting of spleen samples collected at 84 dpi from mice that had been IP-inoculated with RML-SBH and immediately received PLR via the IP route once a week for eight weeks from dpi 1. The average PrP^Sc^ level of the PLR-administrated group was significantly lower than that of the control group that received PBS (Fig. 2). This suggests that PLR effectively suppressed prion propagation and/or PrP^Sc^ accumulation in the spleen at the asymptomatic stage before prions spread to the CNS, proposing a possible manner for modifying the course of prion disease in mice.

### PLR extends the incubation period of mice infected with prions

To evaluate the *in vivo* efficacy of PLR in a mouse model of prion disease, the time period to clinical onset after both RML-SBH inoculation and PLR administration *via* the IP route was measured (Table S1). The prion-inoculated mice that received PLR exhibited a significant extension of the incubation period compared with the control group (Table 1, Fig. S1). In the suppression experiment in which PLR was administered once a week for eight weeks beginning at dpi 1, the incubation period was prolonged more than 16 days. In the prevention experiment in which PLR was administered only once a week for two weeks prior to prion infection and administration ceased after prion inoculation, the incubation period was extended 21 days. These results indicate that PLR effectively suppresses prion disease *in vivo* and could be a useful prophylactic approach.

**Table 1:** Incubation periods in prion-infected mice that received PLR.

Experiments	Treatment		
	Incubation period mean ​± ​SEM (day)	n/n_o_	*p-*value
**Suppression experiment**			
**PBS**	189.5 ​± ​1.5	4/4	
**PLR**	205.9 ​± ​2.0	8/8	0.000319∗∗∗
**Prevention experiment**			
**PBS**	189.0 ​± ​1.7	8/8	
**PLR**	210.0 ​± ​1.9	8/8	0.000041∗∗∗

PBS, phosphate buffered saline (vehicle); PLR, poly-l-arginine; SEM, standard error of the mean; n, number of mice with prion disease; n_o_, number of mice in the group. Log-rank test was used for statistical analysis. ∗∗∗, *p* ​< ​0.001.

### PLR alleviates prion-associated pathological deterioration in mouse brains

**Fig. 3:**
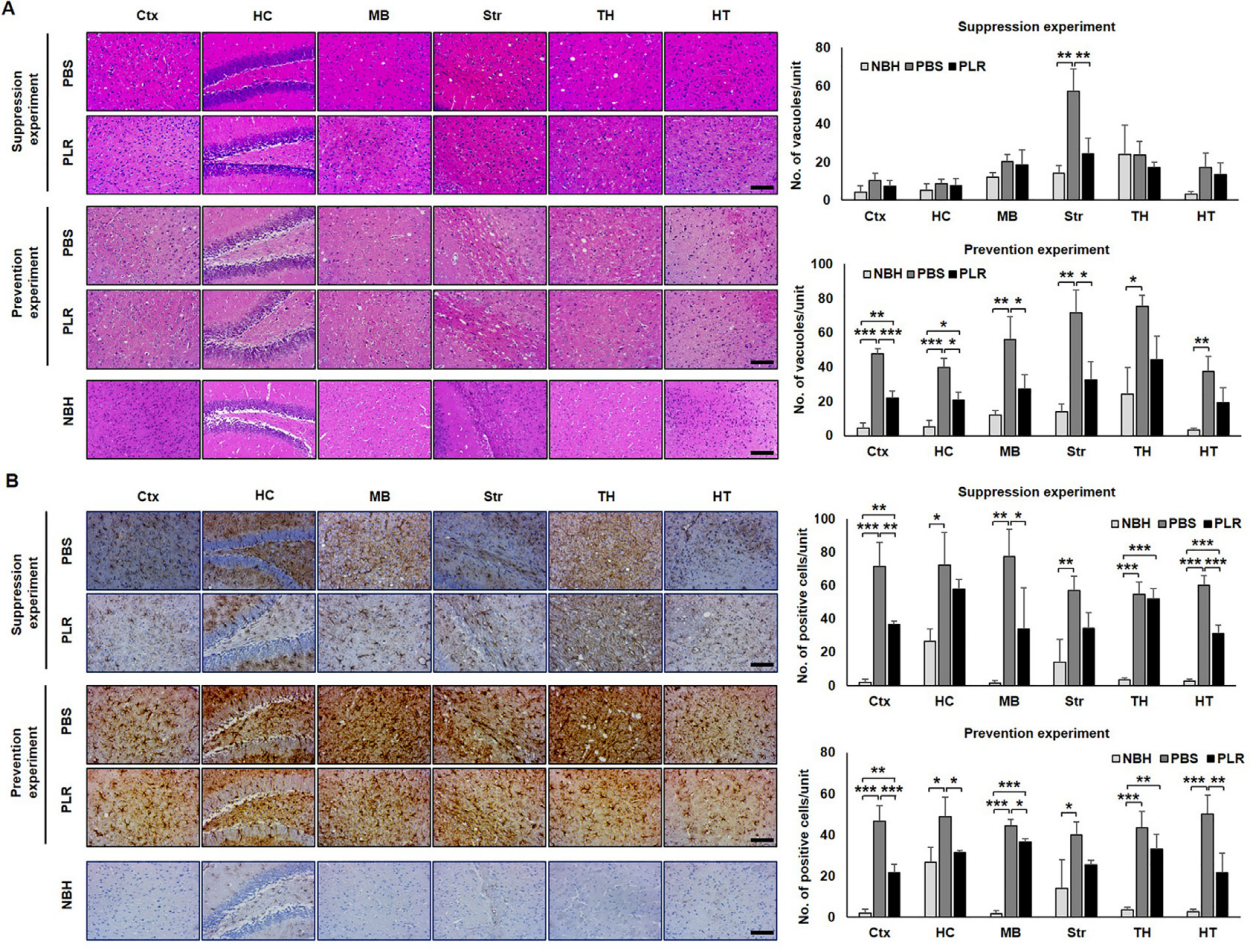
**Pathological differences of prion-infected, PLR-administered mouse brains.** Groups of wild-type mice were IP inoculated with RML-SBH and IP administered PLR once a week for eight weeks in the suppression experiment or IP administered with PLR once a week for two weeks prior to RML-SBH IP inoculation for the prevention experiment. The PBS vehicle was administered in an identical manner. Mock-infection group (NBH, light gray bar); PBS vehicle group (dark gray bar); 25 ​mg/kg PLR group (black bar). The brain sections of mice with clinical onset (*n* ​= ​3) were used for analysis. (A) H&E staining. The number of vacuoles within an arbitrary unit area (equivalent to 0.245 ​mm^2^) was counted repeatedly at different sites (*n* ​= ​3) of each brain section. (B) Immunohistochemistry of GFAP. The spots positively immuno-stained for GFAP expression were counted repeatedly at different sites (*n* ​= ​3) of each brain section. The mean counts of vacuoles and GFAP-positive spots were plotted with their standard deviations. ∗, *p* ​< ​0.05; ∗∗, *p* ​< ​0.01. Ctx, cerebral cortex; HC, hippocampus; MB, mid-brain; Str, striatum; TH, thalamus; HT, hypothalamus. Scale bar: 100 ​μm.

**Fig. 4:**
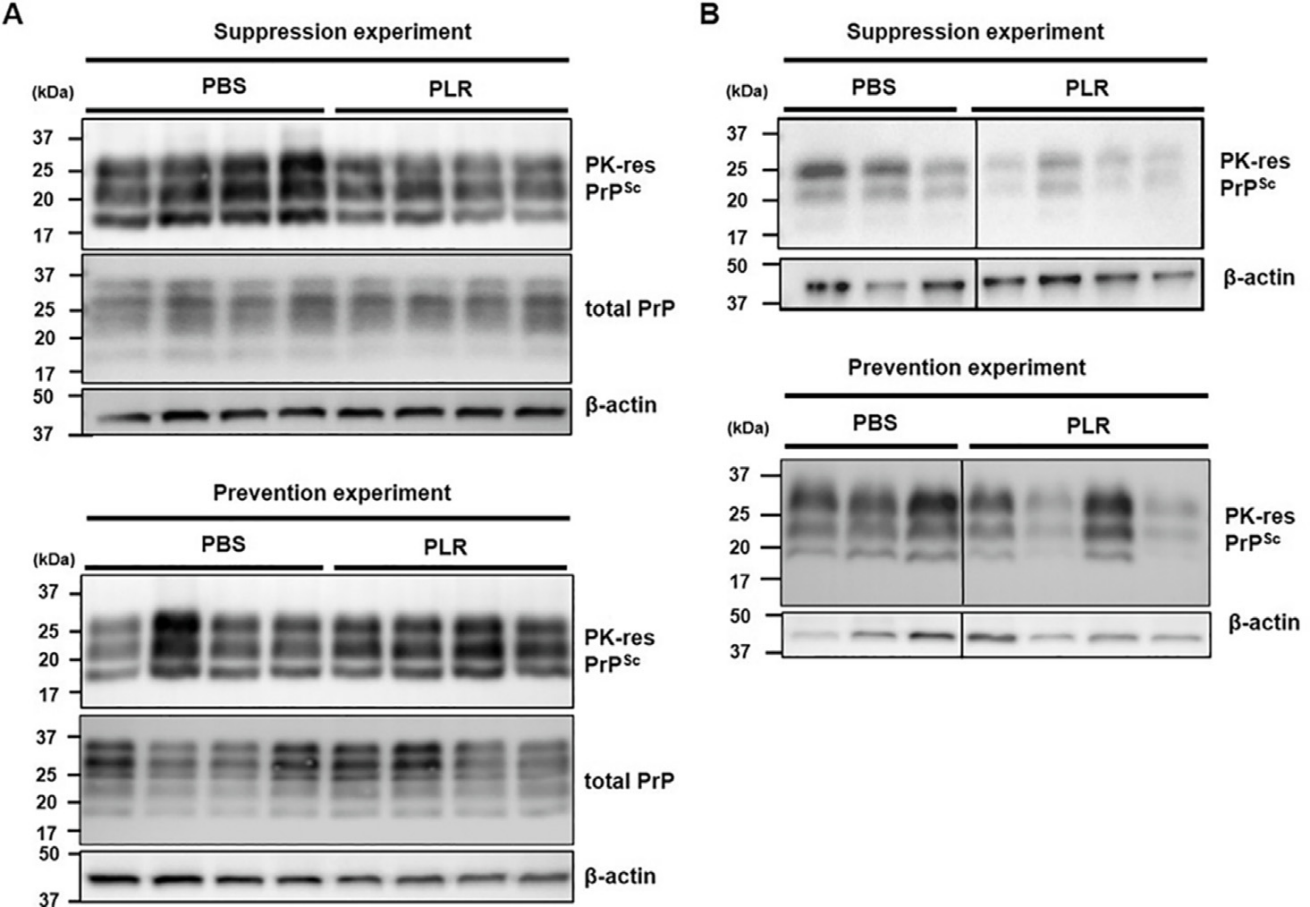
**Western blots of PrP**^**Sc**^**in the brains and spleens of prion-infected, PLR-administered mice.** Groups of wild-type mice were IP inoculated with RML-SBH and IP administered 25 ​mg/kg PLR once a week for eight weeks for the suppression experiment or IP administered PLR once a week for two weeks prior to RML-SBH IP inoculation for the prevention experiments. PBS vehicle was administered in the controls in an identical manner. The whole brain (*n* ​= ​4) and spleen (*n* ​= ​3–4) were collected from each individual mouse with clinical onset of prion disease. Brain and spleen homogenate (10 ​% w/v) was prepared in PBS-2% Sarkosyl, PK digested, and analyzed by western blotting using anti-PrP antibody 6D11. PK-res, PK-resistant. (A) Western blots of PrP^Sc^ and total PrP in mouse brains. (B) Western blots of PrP^Sc^ in mouse spleens.

The efficacy of PLR was confirmed by judging the degree of neuropathological alterations, such as spongiosis and astrogliosis, the representative pathological features of prion disease. PLR reduced the level of vacuolation in the brains of PLR-administered mice, although the effect varied among the brain regions (Fig. 3A). In the suppression experiment, vacuolation was significantly decreased by PLR in the striatum of mouse brains, while reduction by PLR was least effective in other brain regions. In the prevention experiment, the numbers of vacuoles in the cerebral cortex, hippocampus, mid-brain, and striatum were significantly lower than those in the corresponding brain regions of control mice (Fig. 3A). In addition, astrogliosis was markedly decreased in the brains of PLR-administered mice. In suppression experiment, the numbers of GFAP-positive cells in a few common regions of the brain, such as the cortex, mid-brain, and hypothalamus, were significantly lower in the PLR-administered group (Fig. 3B). The cortex, hippocampus, mid-brain, and hypothalamus also displayed a substantial reduction of GFAP-positive cells in PLR-administered mice in the prevention experiment. These data indicate that PLR administration can suppress both spongiosis and astrogliosis in prion-infected brains. However, western blots of PK-resistant PrP^Sc^ in the homogenates of whole brains showed that PrP^Sc^ levels remained unchanged in mouse brains of the PLR-administered groups compared with mouse brains of the control group (Fig. 4A). This indicates that the level of PrP^Sc^ in mouse brains collected at the terminally advanced disease stage did not reflect a quantitative difference that could be measured in terms of PLR efficacy. Interestingly, western blots of PK-resistant PrP^Sc^ in the homogenates of spleens showed that PrP^Sc^ levels were lower in the PLR-administered groups compared with the control group, although they varied among some individual mice (Fig. 4B), suggesting a role of spleen for PLR effect.

### PLR blocks PrP^Sc^ accumulation in splenic white pulp

**Fig. 5:**
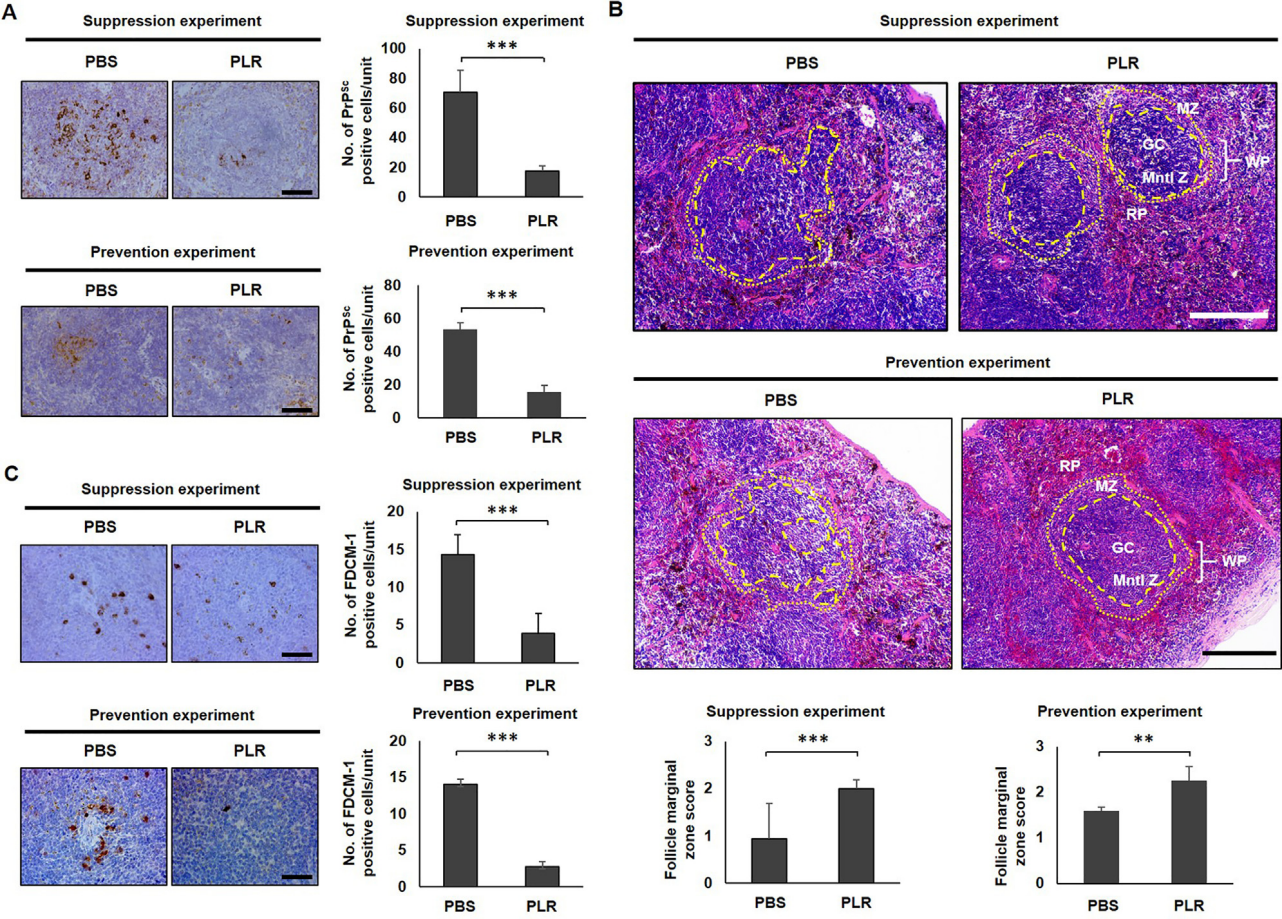
**Pathological differences of prion-infected, PLR-administered mouse spleen.** Groups of wild-type mice were IP inoculated with RML-SBH and IP administered 25 ​mg/kg PLR once a week for eight weeks for the suppression experiment or IP administered PLR once a week for two weeks prior to RML-SBH IP inoculation for the prevention experiment. PBS vehicle was administered in an identical manner. The spleen (*n* ​= ​3) was collected from each individual mouse with clinical onset of prion disease. (A) Representative immunohistochemistry images of PrP^Sc^. The spleen section was immuno-stained for PK-resistant PrP^Sc^. The number of PrP^Sc^-positive cells was counted from randomly chosen splenic follicles (*n* ​= ​3/spleen) of individual mice and plotted with the mean and its standard deviation. Scale bar, 50 ​μm. (B) Representative H&E staining images of splenic follicles and MZ distortion scores. The spleen section was H&E stained, and histological differences were compared. Micrographs with 10× ​magnification was optically magnified and the boundaries between the mantle zone (Mntl Z) of the white pulp (WP) (discontinuous lines) and marginal zone (MZ) and between MZ and the red pulp (RP) (dotted line) were indicated. MZ deformation was scored from multiple follicles (*n* ​= ​6/spleen). The score plots show the means and standard deviations. Scale bar, 50 ​μm. (C) Representative immunohistochemistry images of FDC-M1. The spleen section was immuno-stained for activated FDCs with FDC-M1 marker expression. The cells positive for FDC-M1 were counted from randomly chosen splenic follicles (*n* ​= ​3/spleen) of individual mice and plotted with means and standard deviations. Scale bar: 50 ​μm ∗, *p* ​< ​0.05; ∗∗, *p* ​< ​0.01; ∗∗∗, *p* ​< ​0.001.

To address the effect of PLR on PrP^Sc^ accumulation in the LRS, the spleens of RML-SBH–infected, PLR-administered mice were collected at the terminal stage of disease when mice exhibited clinical signs. IHC analysis of PrP^Sc^ revealed markedly lower accumulation in the white pulp, in which the lymphoid cells reside, in both suppression and prevention experiments (Fig. 5A and Fig. S2). This suggests that PLR efficiently prevents the accumulation of PrP^Sc^ in the white pulp of spleens, and that the cells residing within the white pulp are involved in the suppression of PrP^Sc^ by PLR.

### PLR prevents splenic MZ distortion caused by prion infection

Because characteristic architectural distortion of the MZ (the interface between the lymphoid white pulp and the non-lymphoid red pulp of the spleen) is a common immune process when exogenous pathogens infect a host [[47], [48], [49], [50]], histological deformations of the MZ were examined in RML-SBH-infected or -uninfected spleens. The spleens of control mice with no prion infection retained discrete borders around the white pulp, MZ, and red pulp, while those of prion-infected mice demonstrated indistinct structural confinement in the corresponding regions (Fig. S3A). The scoring of this alteration (MZ scores) demonstrated a significant decrease in architectural integrity of MZ in the spleens of prion-infected mice (Fig. S3B). Next, the effect of PLR on MZ distortion in the spleens of RML-SBH-infected mice was examined. PLR administration prevented MZ destruction, showing less deformation of boundary between the white pulp and MZ, and an increase of MZ scores in both suppression and prevention experiments (Fig. 5B and Fig. S4). These data suggest that PLR impedes architectural alteration of splenic MZ, which is closely associated with PrP^Sc^ accumulation.

### PLR hinders prion-associated FDC activation in mouse spleens

FDCs within the white pulp foster maturation of immune cells in the spleen and play a pivotal role in PrP^Sc^ propagation, accumulation, and spread to the CNS [18,51]. To address FDC activation by prion infection, the expression of FDC-M1 antigens, also known as milk fat globule-EGF factor 8 [52], in and around FDCs was investigated in the spleens of mice with or without RML-SBH infection. Western blot analysis followed by densitometry revealed that expression of FDC-M1 antigens was highly upregulated in the spleens of prion-infected mice (Fig. S5). IHC analysis showed that the stained spots representing activated FDCs that expressed the FDC-M1 antigen were concentrated around the arteriole and scattered within the germinal center of the splenic white pulp of prion-infected mice, but their numbers and intensity were dramatically decreased by PLR in both suppression and prevention experiments (Fig. 5C and Fig. S6). These data suggest that PLR hampers the activation of FDCs involved in prion propagation and PrP^Sc^ accumulation in the spleen.

### PLR-mediated deactivation of FDCs decouples the association of PrP^Sc^ with FDCs in splenic white pulp

**Fig. 6:**
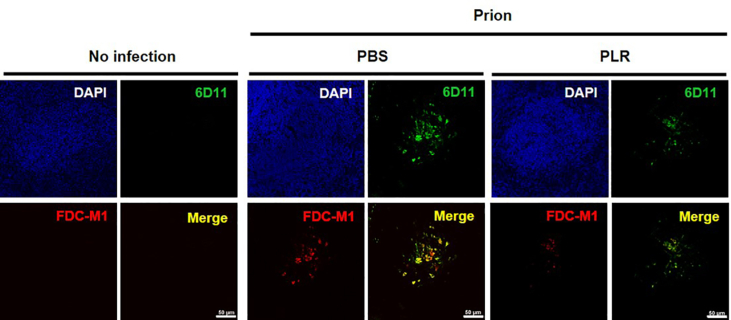
**Immunofluorescence micrographs of PrP**^**Sc**^**and FDC-M1 antigen in splenic white pulps of prion-infected, PLR-administered mice.** The mice were IP inoculated with RML-SBH and IP administered 25 ​mg/kg PLR once a week for eight weeks. The PBS vehicle was administered in an identical manner. The splenic white pulps of mice with clinical onset of prion disease from each group (no infection, no prion inoculated; PBS, prion infected but no PLR administered; PLR, prion infected and PLR administered) were immunofluorescence stained. Images obtained by Z-stacking. Blue, DAPI for nucleus; green, PrP^Sc^ fluorescence with anti-PrP 6D11; red, activated FDC fluorescence with anti-FDC-M1 antibody; yellow, colocalization of PrP^Sc^ with activated FDC (merge). Scale bar, 20 ​μm.

The effects of FDC deactivation by PLR on the association of PrP^Sc^ with FDCs were examined using double-IF microscopy. Similar to the IHC results, PrP^Sc^ accumulation (green fluorescence) and FDC-M1 expression representing FDC activation (red fluorescence) did not appear in the splenic white pulp of uninfected mice, while both occurred in those of RML-SBH–infected mice (the “no-infection” and “PBS” groups in Fig. 6 and Fig. S7). However, FDC-M1 signals were barely detectable, and PrP^Sc^ signals were significantly decreased in the splenic white pulp of PLR-administered, RML-SBH–infected mice (the “PLR” group in Fig. 6 and Fig. S7). In the superimposed fluorescence images, co-localization (yellow fluorescence) of PrP^Sc^ and activated FDCs expressing FDC-M1 antigens in the splenic white pulp of RML-SBH-infected mice was dramatically decreased in PLR-administered, RML-SBH–infected mice (the “PBS” and “PLR” groups in Fig. 6). These results suggest that PLR counteracts activation of FDCs that contribute to the association and proliferation of PrP^Sc^ in the splenic white pulp, resulting in inefficient PrP^Sc^ proliferation and reduced co-localization of PrP^Sc^ and FDC-M1 antigen fluorescence.

## Discussion

Prion diseases are fatal and currently incurable. As with other disorders, the ideal aim of treatment is recovery from the disease state to a normal condition. Such an approach is generally practiced using therapeutic agents and treatment can usually begin after a patient is diagnosed. However, early diagnosis of prion diseases has proven to be difficult, such that the disease is declared at the very late stage when the brain damage is irreversibly advanced. No agents have been shown to recover patients to normal, but a small number of agents produced minor improvements in isolated cases during human trials [30,31,[53], [54], [55]]. Because lesions worsen as prion diseases progress, opportunities to modify the course of the disease are rare if treatment begins at the late phase of disease progression. If treatment begins early, remission can be expected, implying that prophylactic treatment could be a useful option for patients with a certain group of prion diseases such as acquired CJDs caused by peripheral prion exposure.

This study was conducted to investigate the *in vivo* efficacy of prophylactic administration of PLR. For this study, PLR was administered *via* a IP route prior to or soon after prion inoculation. The *in vivo* model used for the study was mice IP inoculated with prions. Prion infection for CJD with known etiologies, such as some vCJD and iCJD, occurs through a non-CNS route, demonstrating that prions infect the hosts not directly in the CNS, but indirectly through the LRS in the periphery. The current investigation with a mouse model designed to reflect aforementioned disease conditions revealed the ability of PLR to modify the course of prion disease. Prophylactic IP administration of PLR delayed the onset of prion disease (Table 1, Fig. S1) and suppressed neuropathological deteriorations in prion-infected mice (Fig. 3). In particular, prevention by administration of PLR once a week for only two weeks prior to prion infection resulted in greater prolongation of incubation periods than suppression by administration of PLR once a week for eight weeks begun at dpi 1. These results provide a foundation for a potential approach to control prion diseases using PLR as a prophylactic rather than a therapeutic agent.

In support of *in vivo* data, *in vitro* infection of cultured cells with prions (RML-SBH) failed following administration of PLR (Fig. 1). This result demonstrates that PLR decreased the infectious tier of prions used to infect cultured cells, suggesting a possibility that the reduction of prion tiers by PLR could facilitate the beneficial effect of PLR observed *in vivo*. Nonetheless, it is unclear how PLR reduces prion infectivity. Biochemical studies in test tubes demonstrated that the efficacy of PLR presented in the current study is not due to destabilization of PrP^Sc^ *via* direct exposure of prions to PLR. Our previous and current studies demonstrated that the stability of PrP^Sc^ was unchanged even if ScN2a cell lysate or the PrP^Sc^-enriched fraction of prion-infected brain homogenate was incubated with PLR in test tubes to allow the direct interaction between PrP^Sc^ and PLR [40] (Fig. S8). Thus, their direct molecular interaction does not appear to explain the effect of reducing prion tiers by PLR. The mechanistic details regarding the decrease of prion infectivity by PLR require further investigations, presumably at the cellular level, whether PLR prevents prion infection in the cells, prion transmission among cells, or prion propagation within the cells.

In addition, it is likely that IP-administered PLR is available in the periphery and hinders prion proliferation in the spleen because the PLR used in this study was too large to pass through the blood-brain barrier. Given that it is unlikely that PLR executes its effect in the brain, inhibition of PrP^Sc^ propagation by PLR in the spleen appears to be more critical than assumed. The speed of disease onset is dependent on PrP^Sc^ proliferation in the LRS, as shown in splenectomized mice [11]. From this perspective, prevention of PrP^Sc^ propagation by PLR in the spleen could delay disease onset in mice. Here, PLR effectively inhibited the accumulation of PrP^Sc^ in the spleens of prion-infected mice during the incubation period before prions spread to the CNS (Fig. 2). This strongly supports the postulate that the spread of PrP^Sc^ to the brain is lessened and/or delayed by the effect of PLR. This is independent of the result showing no difference in PrP^Sc^ accumulation in the end-stage brains of PLR- or vehicle-received mice (Fig. 4A). Although disease onset was delayed, the prion-infected mice that received PLR developed prion disease and steadily accumulated PrP^Sc^ in the brain, eventually reaching similar levels found in the control brains.

These findings suggest that PLR exerts its anti-prion activity by modulating the LRS that functions in initial prion proliferation in the spleens of hosts. Because FDCs are essential LRS cells involved in prion proliferation [18,21,56,57], their activation and PrP^Sc^ proliferation in the spleen were investigated in the present study, and PLR was found to repress those cellular and molecular events (Fig. 4, Fig. 5, Fig. 6, Fig. S2, S4 and S6). Similar to what occurred in the spleen during the asymptomatic period (Fig. 2), PrP^Sc^ accumulation was decreased by PLR in the mouse spleens collected at the terminal stage of prion disease (Fig. 4, Fig. 5A, and Fig. S2). Interestingly, PrP^Sc^ accumulation in the white pulp of the spleen was dramatically reduced. The white pulp is the site where FDCs proliferate and release PrP^Sc^ [19,58,59]. Separately, FDC activation facilitated by prion infection was down-regulated by PLR in the white pulp of the spleen. The expression of FDC-specific FDC-M1 antigen was higher in the spleen after prion infection, but it was lowered by PLR (Fig. 5C, and Fig. S6). This corresponds to the PLR-facilitated reduction of increased FDC-M1 IF in the spleens of prion-infected mice, indicating modulation of FDC activation by PLR (Fig. 6 and Fig. S7). Most importantly, co-IF of colonized PrP^Sc^ and activated FDCs by prion infection was not maintained in splenic white pulp by PLR (Fig. 6). This suggests that PLR either prevents FDC activation or switches off activated FDCs such that they are no longer able to associate with and propagate PrP^Sc^. These results strongly suggest that preemptive suppression of FDC activation by PLR before infection is sufficient to delay the onset of prion disease. Prolongation of the incubation period by PLR can be attributed to the reduction of PrP^Sc^ proliferation in the FDCs and the delay of PrP^Sc^ transmission to the CNS from the spleen. Because prion spread is closely associated with the spleen, and the onset of prion disease requires a complex and lengthy processes of prion migration to the brain, clarification of the role of PLR in the spleen and elucidation of the underlying cellular mechanism are valuable for intervening in disease progression. This study represents an example of such research.

In conclusion, PLR can effectively modify the course of prion disease *in vivo*, demonstrating improved neuropathology. The effect of PLR is realized in the spleen by suppressing PrP^Sc^ propagation, which can impair prion transmission to the brain. Specifically, PLR-mediated deactivation of prion-activated FDCs in the spleen correlates with decreased PrP^Sc^ propagation and decoupling of FDCs with PrP^Sc^. This study indicates the *in vivo* prophylactic efficacy of PLR to control prion disease and elucidates the underlying cellular mechanism. It provides a valuable tool for combating prion disease, suggesting the possibility of controlling prions in the periphery.

## Author contributions

The study was conceived of and designed by Sungeun Lee and Chongsuk Ryou. The study was supervised by Chongsuk Ryou. Sungeun Lee, Jieun Kim, Yoonjeong Lee, Miryeong Yoo, Jaehyeon Kim and Hyun Joo Sohn prepared materials, performed experiments, and collected and analyzed data. The first draft of the manuscript was written by Sungeun Lee, and all authors commented on subsequent versions of the manuscript. The manuscript was finalized by Chongsuk Ryou. All authors read and approved the final manuscript. The study was financially supported by grants.

## Data availability

The datasets generated and/or analyzed in the course of this study are not publicly available due to the lack of a public repository but are available from the corresponding author on reasonable request.

## Ethical approval

The animal experiments were performed under the approval of the Institutional Animal Care and Use Committee at Hanyang University (2017-0158A), Korea.

## Consent for publications

All authors have read and approved the manuscript and supplied consent for publication.

## Declaration of competing interest

The authors declare that they have no known competing financial interests or personal relationships that could have appeared to influence the work reported in this paper.
