# Supplementary material for: Delayed progression of prion disease in mice by polyarginine-facilitated prevention of PrPSc propagation in the spleen
Source: Neurotherapeutics. 2025 Feb 26;22(3):e00560. doi: 10.1016/j.neurot.2025.e00560 (PMC12047483; doi:10.1016/j.neurot.2025.e00560)
Supplement: Multimedia component 1 [file mmc1.docx]

**Supplementary Data**

**Delayed progression of prion disease in mice by polyarginine-facilitated prevention of PrP^Sc^ propagation in the spleen**

**Authors**

Sungeun Lee^1^, Jieun Kim^1^, Yoonjeong Lee^1^, Miryeong Yoo^1^, Jaehyeon Kim^1^, Hyun Joo Sohn^2^, Chongsuk Ryou^1,^*

**Affiliations**

^1^ Department of Pharmacy, College of Pharmacy, and Institute of Pharmaceutical Science & Technology, Hanyang University ERICA

55 Hanyangdaehak-ro, Ansan, Gyeonggi-do, 15588, Republic of Korea

^2^ Foreign Animal Disease Division, Department of Animal and Plant Health Research, Animal and Plant Quarantine Agency

177 Hyeoksin 8-ro, Gimcheon-si, Gyeongsangbukdo, Republic of Korea 39660

***corresponding author**: 55 Hanyangdaehak-ro, Ansan, Gyeonggi-do, 15588, Republic of Korea. Tel: +82-31-400-5811; Fax: +82-31-400-5958; email: cryou2@hanyang.ac.kr

**Running title:** Modulation of prion disease by polyarginine

**Table S1. Experimental design for *in vivo* efficacy tests**


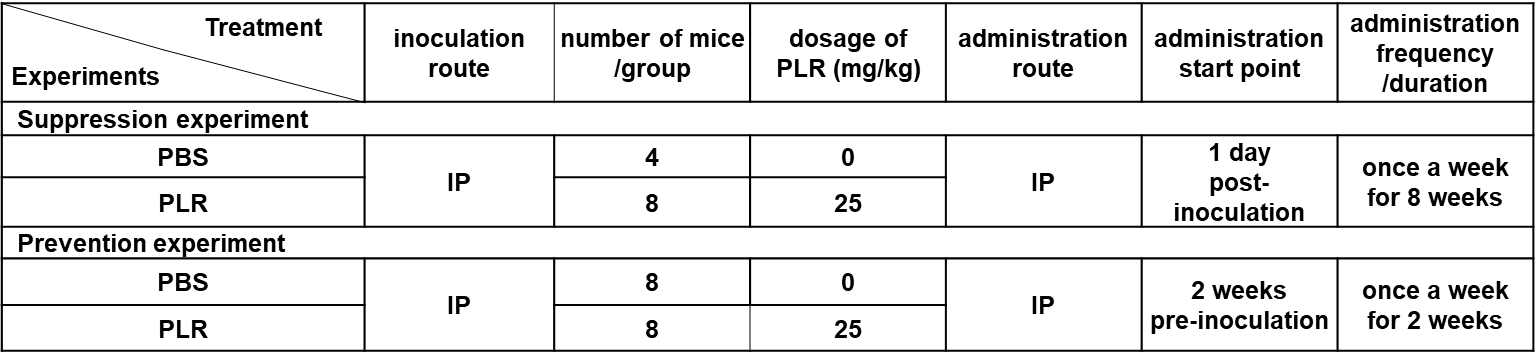
PBS, phosphate buffered saline (vehicle); PLR, poly-L-arginine; IP, intraperitoneal.

**
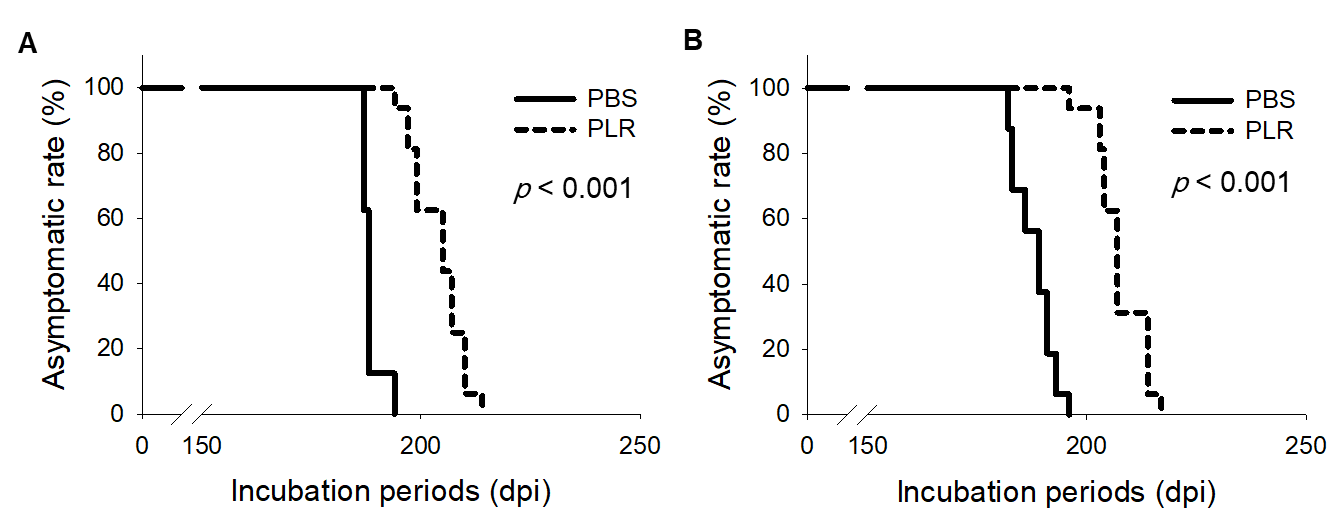
**

**Figure S1. Survival curves of prion-infected mice that received PLR.** Groups of wild-type mice were IP inoculated with RML-SBH and IP administered 25 mg/kg PLR once a week for eight weeks for the suppression experiment (A) or IP administered PLR once a week for two weeks prior to RML-SBH IP inoculation for the prevention experiments (B). PBS vehicle was administered in the controls in an identical manner. PLR-administered groups (discontinuous line) were compared to vehicle-ad mistered group (solid line). The mean incubation period, attack rate, and p-value were presented in Table 1. Difference between groups was statistically analyzed using log-rank test and p-value was smaller than 0.001. dpi, days post-inoculation.


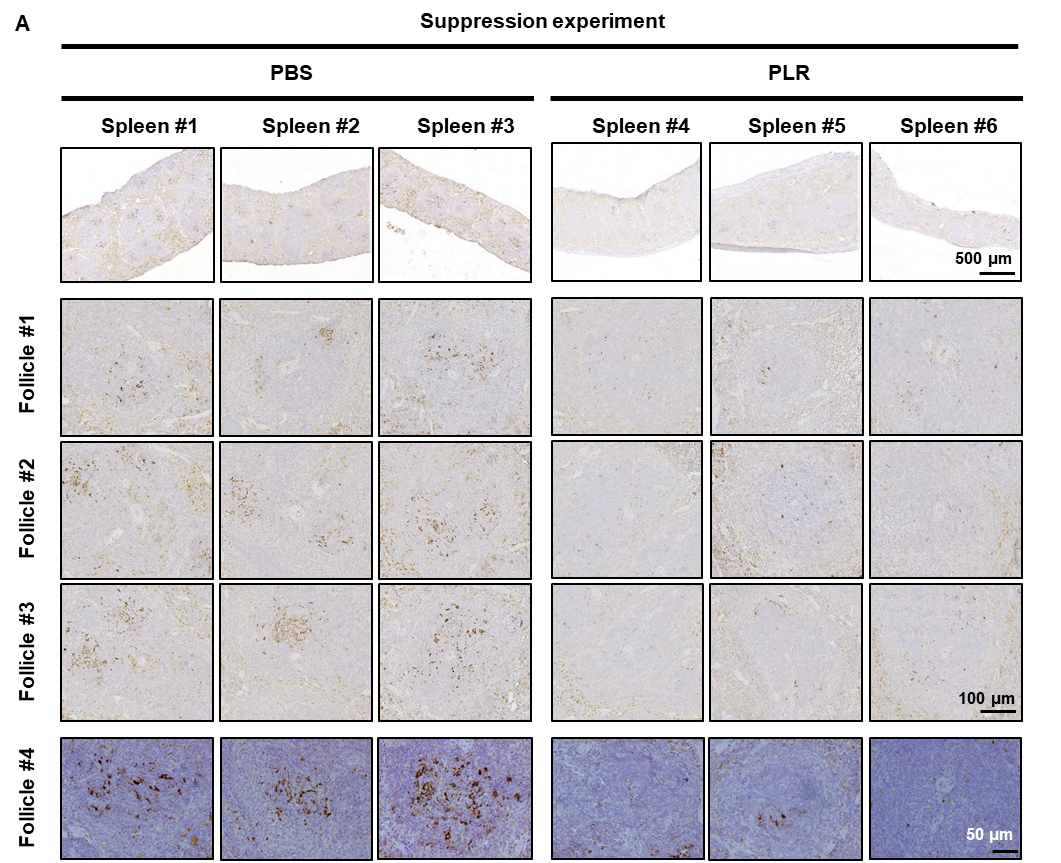


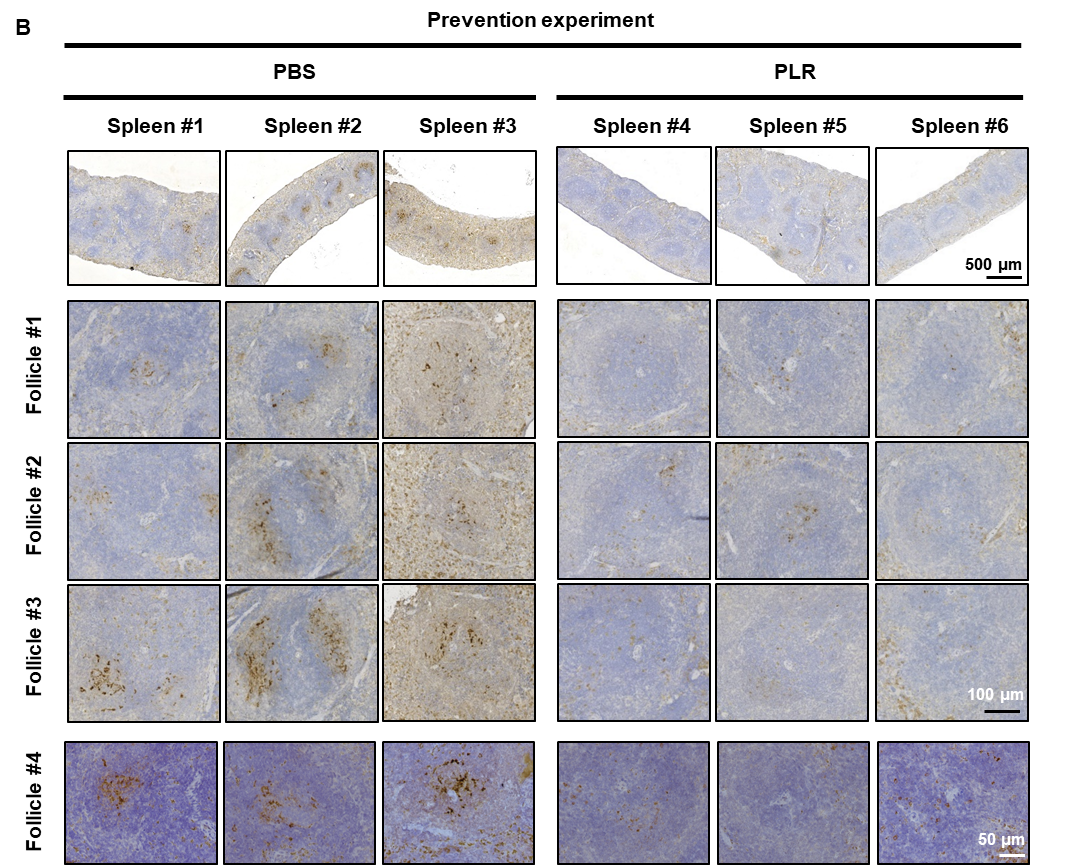


**Figure S2. Immunohistochemistry of PrP^Sc^ in spleens of prion-infected, PLR-administered mice.** Groups of wild-type mice were IP inoculated with RML-SBH and IP administered 25 mg/kg PLR once a week for eight weeks for the suppression experiment (A) or IP administered PLR once a week for two weeks prior to RML-SBH IP inoculation for the prevention experiments (B). The spleen transection slices of individual mice (n = 3) were immuno-stained for PK-resistant PrP^Sc^. Then, splenic follicles (n = 4/spleen) were randomly chosen for presentation. The low power images of spleen and mid-power images of follicles (#1-3) were acquired by Zeiss Axioscan Z1 tissue scanner. The high power images of follicle #4 (the bottom row) were acquired by Nikon ECLIPSE Ti microscope.


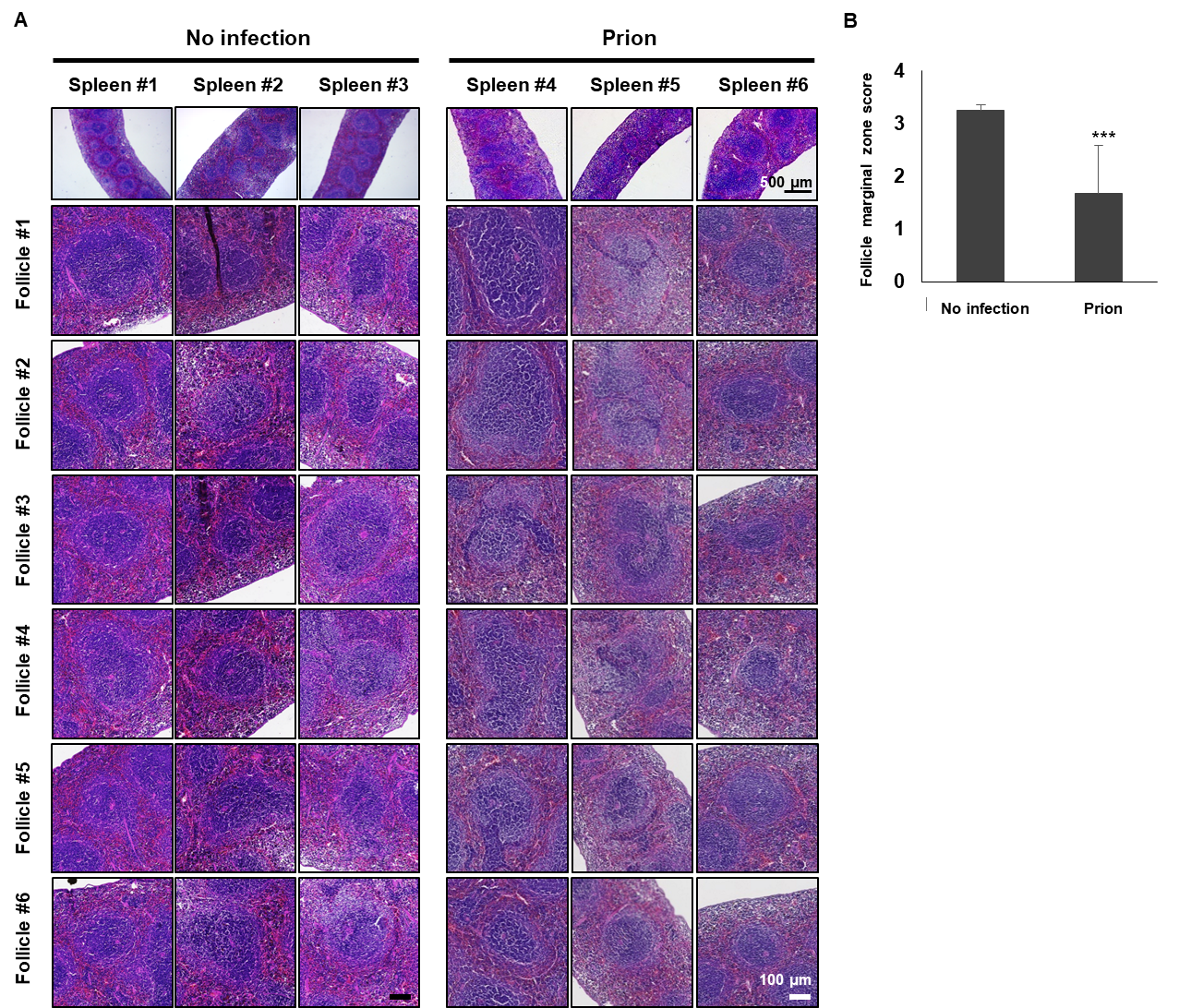


**Figure S3. H&E stains of splenic follicles and surrounding MZ from mock- and prion-infected mice.** Spleens were collected from prion-infected mice with clinical signs at the terminal stage and from age-matched control mice with no prion infection (n = 3/group). (A) H&E stains of spleen and splenic follicles (n = 6/spleen). The low power images of spleen were acquired by Zeiss Axioscan Z1 tissue scanner. The mid-power images of follicles (#1-6) were acquired by Nikon ECLIPSE Ti microscope. (B) MZ score plot. H&E-stained splenic follicles were scored for pathological advancement (MZ deformation) at multiple sites (n = 6) from each mouse spleen (n = 3). ***, *p* < 0.001.


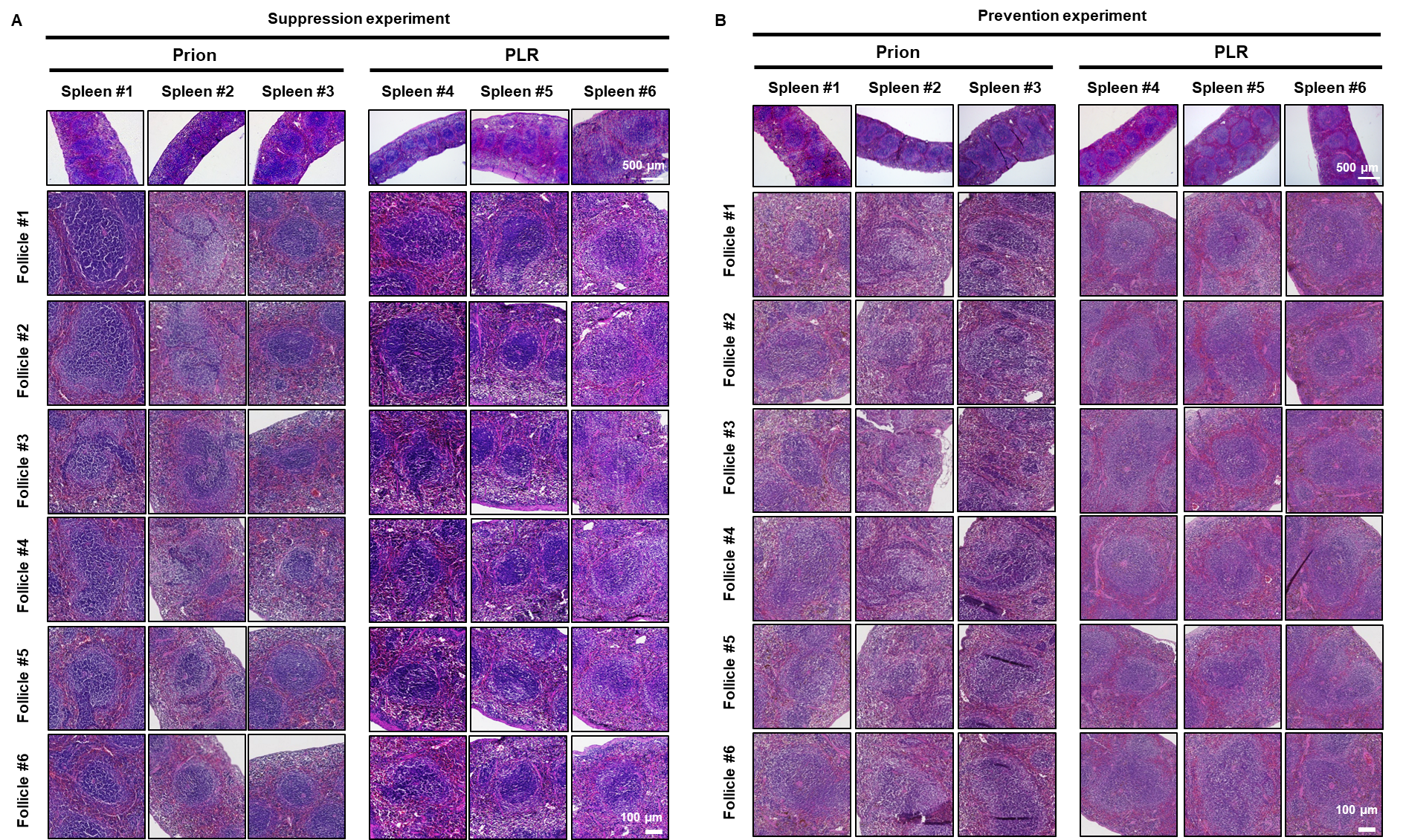


**Figure S4. H&E stains of spleens, splenic follicles and surrounding MZ of prion-infected, PLR-administered mice.** Groups of wild-type mice were IP inoculated with RML-SBH and IP administered 25 mg/kg PLR once a week for eight weeks for the suppression experiment (A) or IP administered PLR once a week for two weeks prior to RML-SBH IP inoculation for the prevention experiments (B). The spleen transection slices of individual mice (n = 3) were H&E stained and the splenic follicles (n = 6/spleen) were randomly chosen for presentation. The low power images of spleen were acquired by Zeiss Axioscan Z1 tissue scanner. The mid-power images of follicles (#1-6) were acquired by Nikon ECLIPSE Ti microscope.


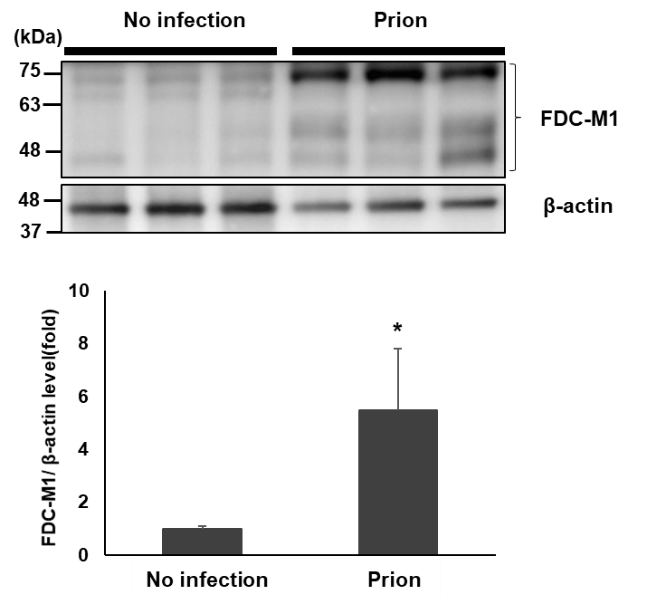


**Figure S5. Western blot of FDC-M1 in RML prion-infected mouse spleens.** Spleens were collected from prion-infected mice with clinical signs at the terminal stage and from age-matched control mice with no prion infection (n = 3 per group). Spleen homogenate (10% w/v) was prepared in PBS, and FDC-M1 expression was immunoblotted. *, *p* < 0.05.


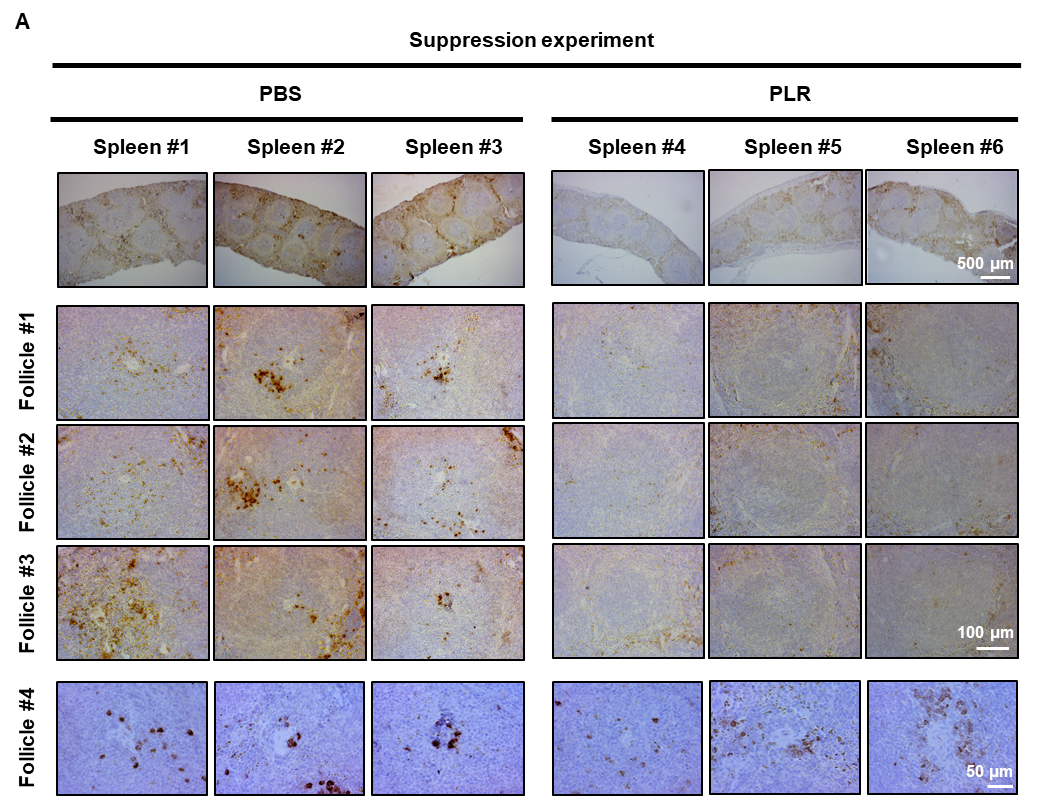


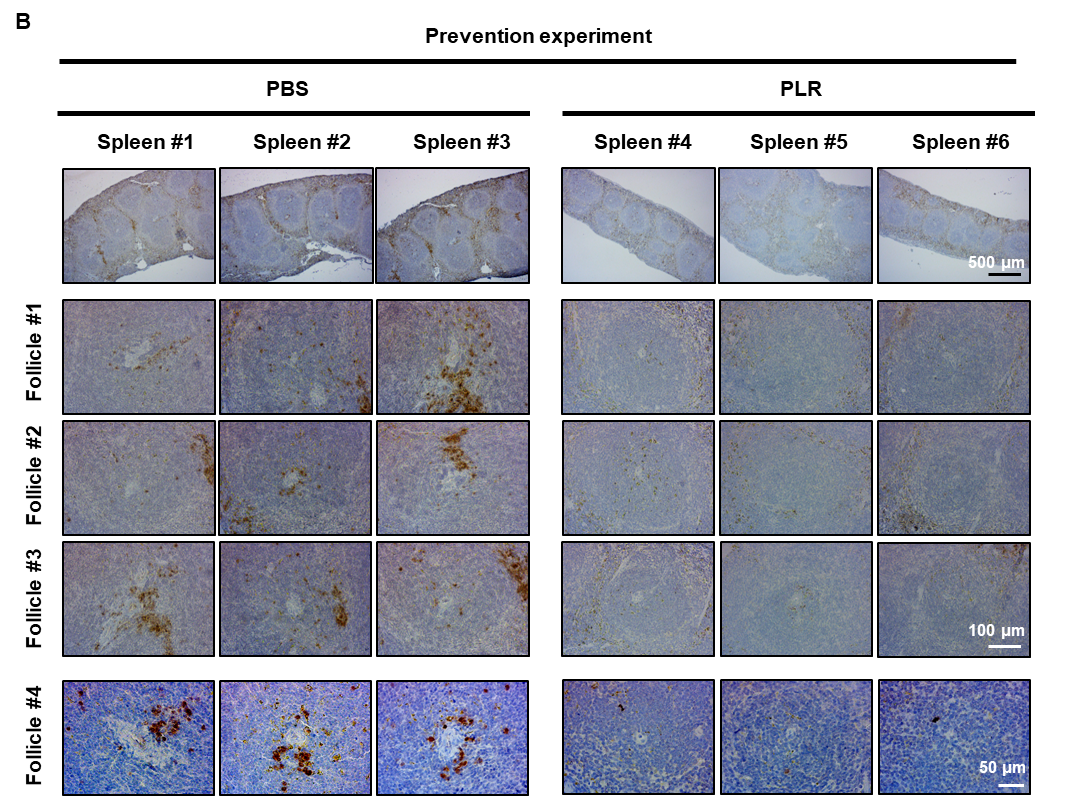


**Figure S6. Immunohistochemistry of FDC-M1 in spleens of prion-infected, PLR-administered mice.** Groups of wild-type mice were IP inoculated with RML-SBH and IP administered 25 mg/kg PLR once a week for eight weeks for the suppression experiment (A) or IP administered PLR once a week for two weeks prior to RML-SBH IP inoculation for the prevention experiments (B). The spleen transection slices of individual mice (n = 3) were immuno-stained for FDC-M1, a marker of FDCs. Then, splenic follicles (n = 4/spleen) were randomly chosen for presentation. The low power images of spleen and mid-power images of follicles (#1-3) were acquired by Zeiss Axioscan Z1 tissue scanner. The high power images of follicle #4 (the bottom row) were acquired by Nikon ECLIPSE Ti microscope.


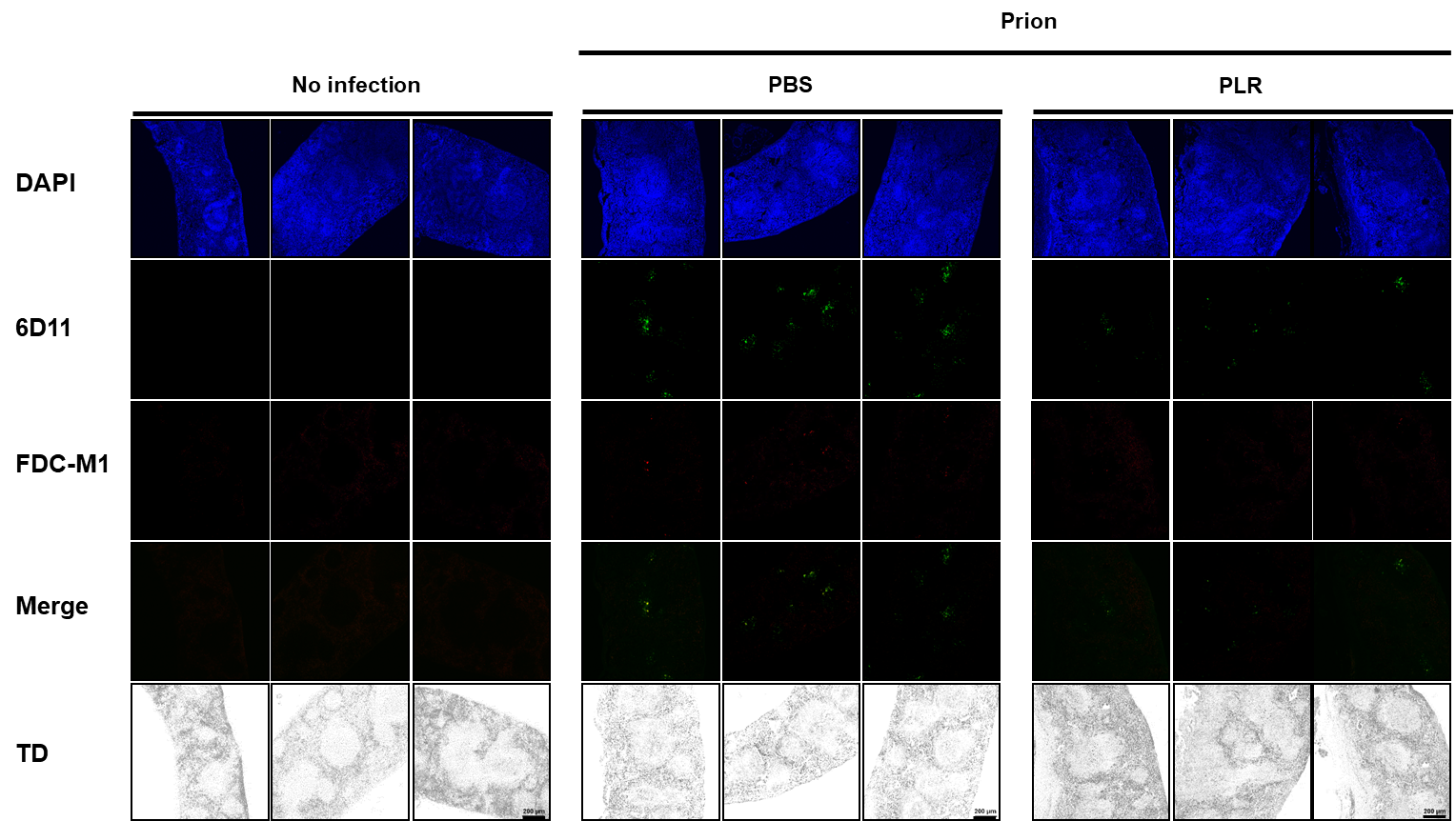


**Figure S7. Immunofluorescence micrographs of PrP^Sc^ accumulation and FDC-M1 antigen expression in the spleens of prion-infected, PLR-administered mice.** The mice were IP infected with prions and IP administered 25 mg/kg PLR. Spleens (n = 3) were collected from mice of each group (no infection, no prion inoculated; PBS, prion-infected but no PLR administered; PLR, prion-infected and PLR administered) at the terminal stage of disease. A low-magnification image of PrP^Sc^ and FDC-M1 distribution in the spleen was obtained using confocal Z-stacking. Blue, DAPI for nucleus; green, PrP^Sc^ fluorescence with anti-PrP 6D11 antibody; red, activated FDC fluorescence with anti-FDC-M1 antibody; yellow, colocalization of PrP^Sc^ and activated FDC (merge); grey, transmitted light detection (TD). Scale bar, 200 μm.


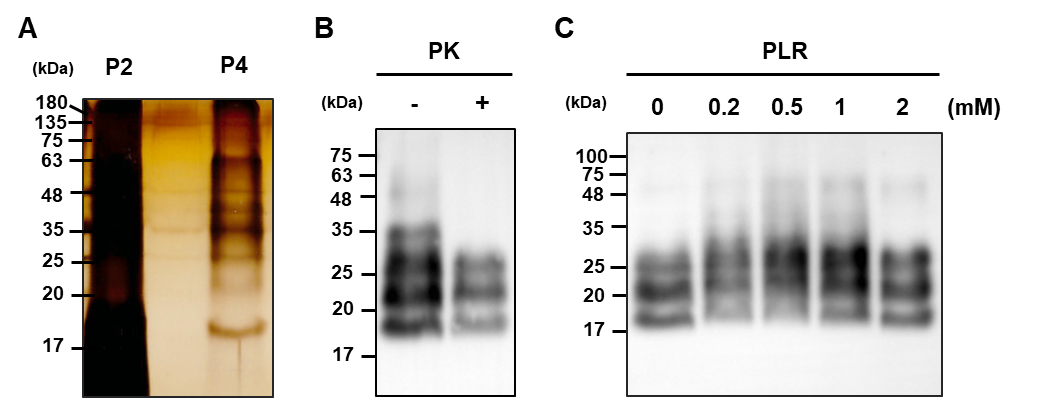


**Figure S8. Effect of PLR on stability of PrP^Sc^.** (A) Silver stain of enriched PrP^Sc^ (P2 and P4 fractions). Enrichment of PrP^Sc^ was performed through repeated high-speed centrifugation (four times) from SBH diluted in a mixture of pronase, sarkosyl, bezonase, sodium phosphotungstic acid, and iodixanol (Wenborn et al., 2015). (B) Western blot of PrP in the P4 fraction before and after PK digestion. (C) Western blot of PrP^Sc^ in the P4 fraction incubated with PLR. The aliquots of P4 fraction were incubated with 0, 0.2, 0.5, 1, and 2 mM PLR38.5 in PBS (pH7.4) for 120 h at 25°C with agitation at 350 rpm. PLR-incubated samples were digested with 20 μg/ml PK and analyzed by western blotting. Highly enriched PrP^Sc^ was not destabilized by PLR, suggesting that the direct interaction of PLR with PrP^Sc^ does not contribute to the anti-prion activity shown in animal models of prion disease.
